# Do Clear Cell Changes in Oral Squamous Cell Carcinoma Warrant It Being Recognised as a Variant?

**DOI:** 10.7759/cureus.25057

**Published:** 2022-05-16

**Authors:** Nurul Inaas Mahamad Apandi, Anand Ramanathan, Siti Mazlipah Ismail, Kannan Ranganathan

**Affiliations:** 1 Department of Oral and Maxillofacial Clinical Sciences, Faculty of Dentistry, Universiti Malaya, Kuala Lumpur, MYS; 2 Department of Craniofacial Diagnostics and Bioscience, Faculty of Dentistry, Universiti Kebangsaan Malaysia, Kuala Lumpur, MYS; 3 Oral Cancer Research and Coordinating Centre, Faculty of Dentistry, Universiti Malaya, Kuala Lumpur, MYS; 4 Oral and Maxillofacial Pathology, Ragas Dental College and Hospitals, Chennai, IND

**Keywords:** prognosis, clinical behavior, diagnosis, variant, clear cell changes, oral squamous cell carcinoma, oral cancer

## Abstract

Histological variants of oral squamous cell carcinoma (OSCC) include verrucous, basaloid, spindle cell, adenosquamous, papillary, and acantholytic types. Clear-cell changes in OSCC are rare.

We report a case of a 65-year-old male Chinese patient who presented with a swelling in the lower-left mandible for three weeks, causing ill-fitting of his lower denture and an ulcer on the floor of the mouth. Histologically, the lesion showed a dense proliferation of malignant tumor cells arranged in islands and sheets consisting of squamoid cells intermixed with signet ring-shaped clear cells. The clear cells were negative for mucicarmine, periodic acid Schiff (PAS), periodic acid Schiff-diastase (PAS-D), and alcian blue (AB). Immunohistochemistry showed the tumor cells were immuno-positive for cytokeratin (CK) and p63, but CK7, CK20, and S100 were immuno-negative. Therefore, a metastatic tumor in the oral cavity was suggested. However, the CT scan did not show any primary tumors in other sites. Histopathologically, the surgical specimen showed signet-ring-shaped clear cells in the stroma with squamoid cells invading the underlying connective tissue from the surface epithelium, suggesting a diagnosis of clear cell changes in OSCC. Follow-up showed recurrent OSCC at the base of the tongue with lymph node metastasis and distant metastasis in the lung.

Only a few cases of clear-cell changes have been reported, with most having a poor prognosis. This case report adds one more case of clear cell changes in the OSCC with a poor prognosis. We reviewed the literature to understand their clinical behavior. Due to the rarity of its (clear cell changes) occurrence, further research is required in order to obtain a better understanding of the clinical behavior and prognosis of these clear cell changes seen in OSCC.

## Introduction

Oral squamous cell carcinoma (OSCC) is the sixth most common cancer in the world [1]. OSCC is a carcinoma with squamous differentiation arising from the mucosal epithelium. It is the most common head and neck neoplasm, accounting for more than 90% of cases. Histopathological presentation is characterized by surface epithelium infiltrating as nests, sheets, and cords into the underlying connective tissue. Grading is based on architectural and cytological features and is divided into, well, moderate, and poor differentiation [2]. The known histological variants include basaloid, spindle cell, adenosquamous, verrucous, papillary, and acantholytic types [3]. The clear cell variant was described by Carmer and Heggeness in 1989 [4]. To the best of our knowledge, only seven cases of clear cell changes in OSCC have been reported previously [5-11]. Thus, in this article, we present another case of clear cell changes seen in OSCC and a review of the literature to understand their clinical behavior and see whether this histopathological feature (clear cell changes) needs to be recognized as a variant of OSCC.

## Case presentation

A 65-year-old male Chinese patient presented with an intraoral swelling in the lower-left premolar-molar region that was present for three weeks. It caused his lower denture to become loose. The growth was gradually increasing in size and bled upon brushing. The patient did not show any signs of weight loss, loss of appetite, or dysphagia. He was hypertensive and was on 5 mg of amlodipine once daily. Sixteen years ago, he had a history of triple vessel disease and a transient ischaemic heart attack. Therefore, he underwent a coronary artery bypass graft and was under regular follow-up. His father had prostate cancer. He was an ex-smoker and quit smoking 10 years ago and had no other current risk habits.

**Figure 1:**
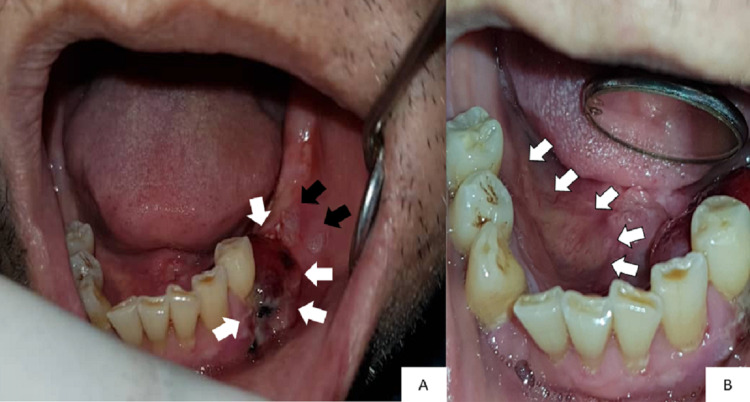
Intraoral photographs show (A) a swelling in the lower-left premolar-molar region (white arrows) and two white patches (black arrows) and (B) an irregular ulcer on the right side of the floor of the mouth (white arrows)

Intraorally, there was a 2 cm x 1.5 cm swelling in the lower-left premolar-molar region, which was edentulous (Figure 1A). The swelling was soft and reddish in color. There were two white patches present posterior to the swelling (Figure 1A). These white patches were non-scrapable. The largest white patch measured 1 cm x 1 cm in size. There was also an irregular ulcer on the right-side floor of the mouth approximately 1 cm x 4 cm in size, with a slightly yellowish bed and erythematous margins on the floor of the mouth (Figure 1B). Incisional biopsies were taken from these sites and sent for histopathological examination.

**Figure 2:**
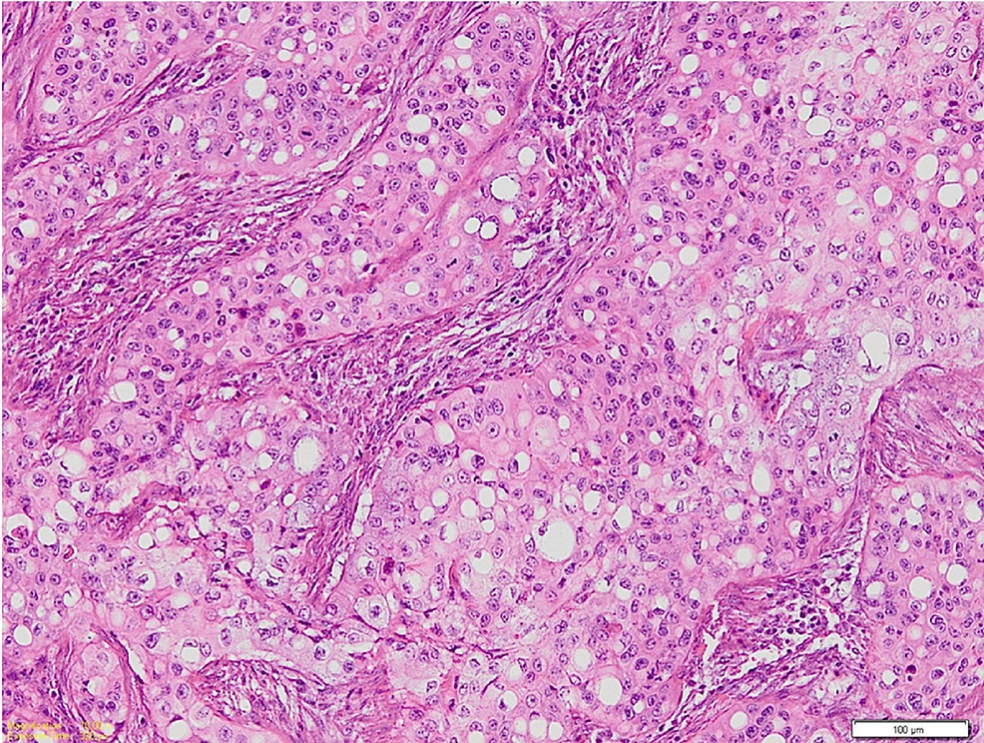
Photomicrograph shows numerous neoplastic cells having signet ring-shaped with clear cytoplasm and eccentrically placed nucleus Stain: H&E; Original magnification: 100X

Microscopic examination of the hematoxylin and eosin (H&E)-stained sections of the biopsy specimens from the swelling and ulcer showed islands and sheets of squamoid cells in the connective tissue stroma not arising from the surface epithelium. They had lobules and a nest of tumor cells having prominent signet ring-shaped cells arranged in an organoid pattern (Figure 2). These signet ring-shaped cells had clear cytoplasm and an eccentrically placed nucleus. The tumor cells showed nuclear and cellular pleomorphism, altered nuclear to cytoplasmic ratio, and numerous mitoses. The H&E-stained sections showed the presence of epithelial hyperkeratosis in the biopsy from the two white patches.

**Figure 3:**
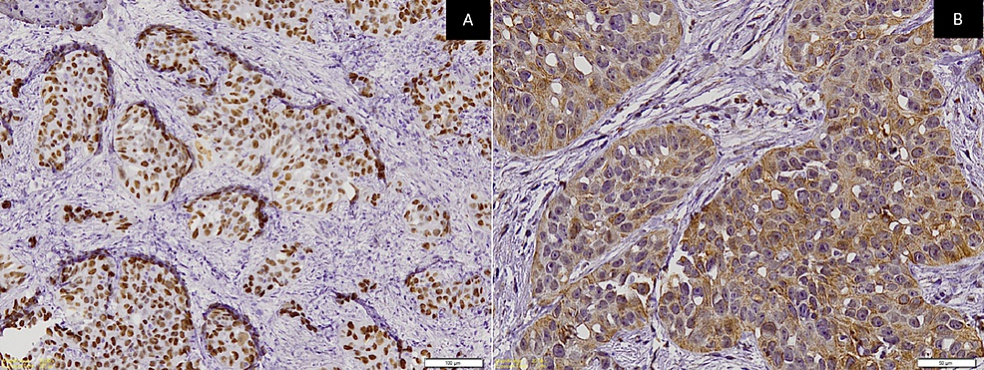
Photomicrographs show neoplastic cells that are immunopositivite for (A) p63 (Original magnification: 100x) and (B) CK (Original magnification: 200x)

**Figure 4:**
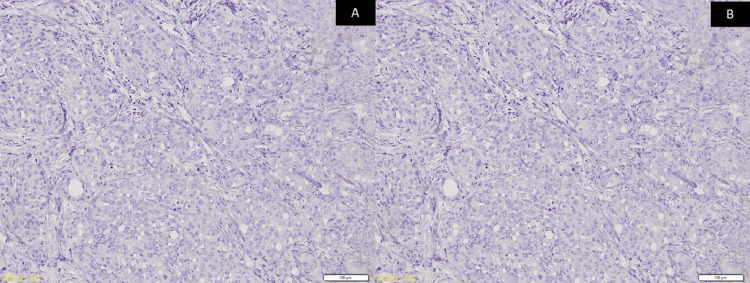
Photomicrographs show neoplastic tumor cells that are immunonegative for (A) CK7 (Original magnification: 100x) and (B) CK20 (Original magnification: 100x)

A panel of immunohistochemistry was done, including p63 and CK, which were immuno-positive for tumor cells (Figures 3A-3B) while CK7 and CK20 were immuno-negative (Figures 4A-4B) along with S100. Special stains were done and the clear cells were negative for mucicarmine, periodic acid Schiff (PAS), periodic acid Schiff-diastase (PAS-D), and alcian blue (AB). Hence, from the incisional biopsies, a diagnosis suggestive of metastatic signet ring cell adenocarcinoma of an occult primary was made.

**Figure 5:**
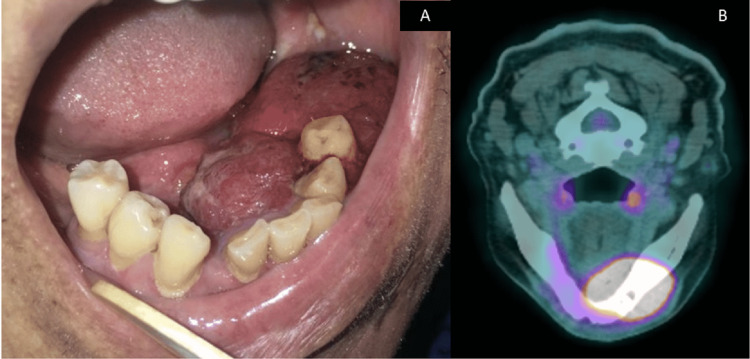
(A) Intraoral photograph shows a large growth involving the left side floor of the mouth extending from the midline to the edentulous molar region. (B) Photograph shows PET-CT image (axial cut) of the primary tumor in the left side body of the mandible PET-CT: positron emission tomography-computed tomography

The attending oral surgeon was informed of the diagnosis, and a computed tomography (CT) scan of the head and neck, thorax, and abdomen was performed. However, there was no evidence of tumors in other areas. This was conveyed to the oral pathologist. Following that, a revised working diagnosis of primary carcinoma of the oral cavity was suggested. Within two weeks, the primary lesion on the left side of the mandible had rapidly increased in size (Figure 5A) when compared to the lesion seen in Figure 1A. The positron emission tomography-computed tomography (PET-CT) image of this lesion is shown in Figure 5B. Under general anesthesia, a left segmental mandibulectomy with wide excision of the tumor was carried out. Bilateral supraomohyoid neck dissection and reconstruction with a free fibula flap were performed.

**Figure 6:**
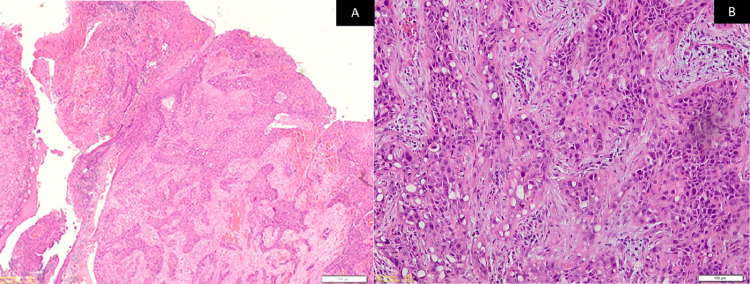
Photomicrograph shows (A) sheets of infiltrating squamoid tumor cells from the surface (Stain: H&E; Original magnification: 20X) and (B) numerous neoplastic cells showing clear cell changes (Stain: H&E; Original magnification: 200X)

The H&E-stained sections of the surgical specimen showed numerous squamoid tumor cells invading the underlying connective tissue stroma from the surface epithelium (Figure 6A). The anaplastic tumor consisted of a dense proliferation of neoplastic cells arranged in islands and sheets consisting of epithelioid tumor cells intermixed with signet ring-shaped clear cells. Tumor cells exhibited vesicular nuclei and faintly eosinophilic to clear cytoplasm with an indistinct cell border (Figure 6B). The tumor islands were seen extending from the surface epithelium into the underlying lamina propria and submucosa. Superficially, the tumor cells were arranged in a squamous cell pattern, but deeper, they were arranged in an organoid pattern. Abnormal mitosis was increased and central necrosis within islands was observed. Special stains for mucicarmine and PAS-D were negative. CK and p63 were again immunopositive, whereas CK7 and CK20 were immunonegative along with S100. The margins were clear of the tumor. Infiltrative bone invasion was seen in the mandible. Neck dissections were all free of tumors from Levels I-IV on both sides. Therefore, a final and definitive diagnosis of moderately differentiated squamous cell carcinoma with clear cell changes having an unusual organoid pattern was made. The pathological stage of the tumor was T4aN0M0 (Stage IVA).

**Figure 7:**
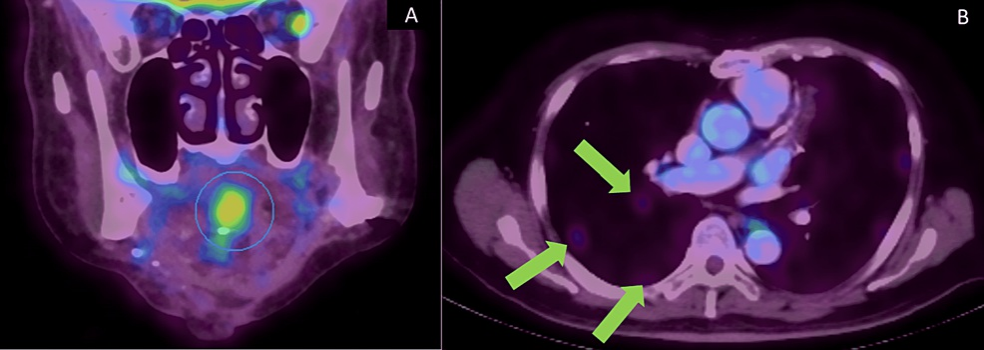
Photograph shows PET-CT image (A) of the recurrent tumor on the base of the tongue (blue circle) and (B) three metastatic nodules in the right lung (green arrows) PET-CT: positron emission tomography-computed tomography

Since the tumor had invaded the mandible, the patient was advised to undergo adjuvant radiotherapy. However, the patient declined to undergo adjuvant radiotherapy. Recurrence occurred in December 2020, which was two years and two months after the treatment of the primary tumor. The new hypermetabolic lesion with ametabolic central was seen in the base of the tongue within the genioglossus muscle measuring 3.9 x 3.3 x 2.8 cm (Figure 7A). Two sub-centimeter hypermetabolic level II nodes on the left side of the neck were also noted. No distant metastasis was noted. Fine needle aspiration cytology (FNAC) of the recurrent lesion was diagnosed as SCC. The patient underwent adjuvant concurrent chemoradiation therapy (CCRT) with carboplatin for five cycles and 70 Gy of 35 cycles of radiation. However, a PET-CT taken in July 2021 showed multiple new lung metastases (Figure 7B). The patient was last reviewed in December 2021. The recurrent SCC at the base of the tongue and the two lymph nodes on the left side of the neck at level II had resolved. The metastatic lung lesions were seen reduced in size.

## Discussion

Seven cases of clear cell changes in OSCC have been reported previously (Table 1) [5-11]. Signet ring cells are defined as cells whereby the nucleus is displaced as a result of compression by a cytoplasmic component [12]. Primary malignant neoplasms with clear cell changes within the oral cavity are linked to salivary gland malignancies and clear cell changes in odontogenic tumors [13]. Clear cell OSCC is an exceptionally rare variant of OSCC. It was first reported in 1980 by Kuo as a hydropic OSCC. The clear cell changes are attributed to extensive hydropic degeneration of neoplastic cells and accumulation of intercellular fluid and not to accumulation of stromal components such as lipid, glycogen, or mucin. Most cases of clear cell OSCC have been reported to occur in the mandible where the clinical presentation is of a nodular mass that may be ulcerated [14].

**Table 1:** Reported cases of clear cell changes in oral squamous cell carcinoma CCRT: concurrent chemoradiation therapy

Author	Year	Age	Gender	Site	Treatment and outcome
Frazier et al [5]	2012	59	Female	Mandibular gingiva	Referred for Oncology consultation, however, defaulted and was lost to follow-up.
Kumar et al [6]	2012	70	Female	Anterior maxilla and right mandible (2 sites)	Surgical excision of the lesion and bilateral supra-omohyoid neck dissection. Unfortunately, the patient succumbed within 2 months of follow-up.
Romanach et al [7]	2014	60	Female	Buccal mucosa extending to the soft palate	Surgical removal with adjuvant radiotherapy. 6 months local recurrence with no regional metastasis and no recurrence after 12 months post second surgery.
Nainani et al [8]	2014	52	Male	Buccal mucosa	Complete excision of primary lesions with bilateral supraomohyoid neck dissection was done. Metastasis to Level Ia was observed. The patient was advised for radiotherapy but declined and succumbed to dissemination disease after 3 months.
Kaliamoorthy et al [9]	2015	35	Female	Tongue and lingual vestibule	Referred to the cancer institute for comprehensive management.
Devi et al [10]	2016	55	Male	Maxillary alveolar ridge	Hemimaxillectomy with left radical neck dissection. Reviewed 5 months post-operation and still undergoing radiotherapy.
Khoury et al [11]	2017	66	Female	Tongue, the floor of the mouth, and the retromolar fossa	The patient underwent subtotal glossectomy, partial pharyngectomy, bilateral neck dissection, and simultaneous anterolateral thigh free flap reconstruction. 3 months after salvage surgery, a chest CT scan indicated metastasis to the left lung. The patient was scheduled for chemotherapy and a potential clinical trial.
Present case		65	Male	Floor of mouth	Wide excision of tumor at left mandible with segmental mandibulectomy along with bilateral extended supraomohyoid neck dissection. Reconstruction was done with a free fibula flap. Recurrence occurred at the base of the tongue 2 years and 2 months after initial treatment with 2 hypermetabolic lymph nodes on the left side of the neck in level II. The patient was given CCRT. Lung metastasis was noted. In the last review, the recurrent lesion on the base of the tongue, the level II lymph nodes, and the lung metastasis were resolved. The lung metastasis was reduced in size in December 2021.

Clear cell in squamous cell carcinoma (SCC) of the skin is divided into three categories: keratinizing (Type I), non-keratinizing (Type II), and pleomorphic (Type III) [15]. Type I is characterized by tumor cells with a clearing of cytoplasm and the nucleus displaced to the periphery, thus leading to a foamy appearance, making it difficult to distinguish from other clear cells such as adipocytes and sebaceous cells. The stromal component is fibrotic with scarce inflammatory infiltrates. Type II distinctively originates from the dermis and has no connection with the overlying epidermis. Tumor cells are arranged in parallel or anastomosing cords separated by compressed fibrous connective tissue and a dense inflammatory infiltrate. Tumor cells exhibit central nuclei with clear cytoplasm and central necrosis may be evident focally in cords. Type III is associated with substantial ulceration. Cytological atypia is evident, and tumor cells display marked nuclear and cellular pleomorphism with dyskeratoses and foci of squamous differentiation. Perineural and vascular invasion are also common findings. Among all three types, none show glycogen or mucin within the tumor cells [15] as in the present case. This coincides with the postulated hypothesis that clear cell OSCC occurs as a result of hydropic degeneration.

Differential diagnosis of clear cell OSCC neoplasm includes a wide range of benign and malignant tumors. The benign tumors of salivary gland origin considered include myoepithelioma and oncocytoma whereby the clear cell composition may predominate in both lesions. In addition, salivary gland malignancies considered include mucoepidermoid carcinoma, acinic cell carcinoma, myoepithelial carcinoma, epithelial-myoepithelial carcinoma, and hyalinizing clear cell carcinoma. As the oral cavity may harbor distant metastatic tumors hence metastatic renal cell carcinoma is also considered. Neoplasm of odontogenic origin to be ruled out include clear cell calcifying epithelial odontogenic tumor; however, this particular variant is quite consistent with the biphasic histomorphological pattern. Along with clear cell changes, odontogenic carcinoma should also be ruled out. All these can best be differentiated from one another by their classical histologic features and designated immunohistochemical markers. Clearing may be due to cytoplasmic clearing in formalin‑fixed sections that are stained with H&E. In our case, artifacts were ruled out as the specimen was immersed in standard fixation of 10% neutral buffered formalin for more than the 24-hour method, and conventional laboratory procedures for processing tissues and staining were done according to the standard operating protocol. Besides that, clear cell components in a lesion may also be manifestations of physiological changes, such as cell organelles scarcity within ductal structures, and accumulation of intracellular substances such as glycogen, lipid, mucin, and zymogen granules.

A panel of immunohistochemistry was done, including p63 and CK, which were positive. Mucoepidermoid carcinoma was ruled out as PAS and mucicarmine were negative and due to the absence of intermediate cells and mucin histopathologically. Acinic cell carcinoma, myoepithelial carcinoma, and epithelial-myoepithelial carcinoma were ruled out as PAS and S100 were negative. The absence of dense fibrotic stroma ruled out hyalinizing clear cell carcinoma and clear cell carcinoma of odontogenic origin. Metastatic renal cell carcinoma was ruled out as CK20 was negative and histologically, it lacked pronounced sinusoidal vascular channels with hemorrhagic foci [16]. However, since CK7 and CK20 were negative, metastatic adenocarcinoma [17], probably from the prostate, was considered, and the same was conveyed to the attending oral surgeon. Moreover, aberrant expression of p63 in prostate adenocarcinoma has been reported [18] and p63 is positive in this case. However, when no primary tumor was found in the CT scans of the head and neck, thorax, and abdomen in this patient, a revised working diagnosis of carcinoma of the oral cavity was suggested. Hence, this case emphasizes the need for close communication between the surgeon and the pathologist, which can facilitate the best treatment for the patient. Later, based on clinical, histopathology, and immunohistochemistry evaluation of the surgical specimen, we concluded the diagnosis as suggestive of moderately differentiated OSCC with an unusual organoid pattern with clear cell changes.

In the literature, seven cases of OSCC with clear cell changes have been reported (Table 1), with one being synchronous [6]. OSCC with clear cells seems to occur predominantly in females (five cases) with a wide age range (35 to 66 years). It affects the subsites, such as the gingiva, buccal mucosa, maxilla, mandible, tongue, and floor of the mouth, in the oral cavity (Table 1). The prognosis was poor in most of the cases, with local recurrence [7], regional lymph node metastasis [8], distant metastasis to the lung [11], and death [6] being reported in one case each. The present case had a recurrence, with lymph node metastasis and distant metastasis to the lungs indicating aggressive behavior and poor prognosis.

## Conclusions

Clear cell changes in OSCC are unusually rare and cause difficulty in diagnosis, as many other tumor entities would have this differentiation. Thus, a meticulous and thorough evaluation of all aspects of clinical, radiological, and histopathological components is required to arrive at a definite diagnosis.

Most of the cases of OSCC showing clear cell changes show poor prognosis including the present case. However, due to the rarity of its occurrence, it is difficult to determine the clinical behavior of OSCC with clear cell changes. Therefore, further research is required in order to obtain a better understanding of the clinical behavior and prognosis of these clear cell changes seen in OSCC. Hence, at this juncture, it is difficult to answer the question. "Do clear cell changes in OSCC warrant it being recognized as a variant?" thus awaiting further evidence.

## References

[1] Warnakulasuriya S (2009). Global epidemiology of oral and oropharyngeal cancer. Oral Oncol.

[2] Li Y, Bai S, Carroll W (2013). Validation of the risk model: high-risk classification and tumor pattern of invasion predict outcome for patients with low-stage oral cavity squamous cell carcinoma. Head Neck Pathol.

[3] Mallick S, Breta M, Gupta SD, Dinda AK, Mohanty BK, Singh MK (2015). Angiogenesis, proliferative activity and DNA ploidy in oral verrucous carcinoma: a comparative study including verrucous hyperplasia and squamous cell carcinoma. Pathol Oncol Res.

[4] Cramer SF, Heggeness LM (1989). Signet-ring squamous cell carcinoma. Am J Clin Pathol.

[5] Frazier JJ, Sacks H, Freedman PD (2012). Primary glycogen-rich clear cell squamous cell carcinoma of the mandibular gingiva. Oral Surg Oral Med Oral Pathol Oral Radiol.

[6] Kumar K, Shetty DC, Wadhwan V, Gupta P (2012). Synchronous oral squamous cell carcinomas with unusual histopathological feature. J Oral Maxillofac Pathol.

[7] Romañach M, Canedo N, Cortezzi E (2014). Clear cell variant of oral squamous cell carcinoma. Oral Surg Oral Med Oral Pathol Oral Radiol.

[8] Nainani P, Singh HP, Paliwal A, Nagpal N (2014). A rare case report of clear cell variant of oral squamous cell carcinoma. J Clin Diagn Res.

[9] Kaliamoorthy S, Sethuraman V, Ramalingam SM, Arunkumar S (2015). A rare case of clear cell variant of oral squamous cell carcinoma. J Nat Sci Biol Med.

[10] Devi A, Kamboj M, Singh V, Singh S (2017). Clear-cell variant of squamous cell carcinoma in maxilla as primary lesion: a rare case. J Oral Maxillofac Pathol.

[11] Khoury ZH, Bugshan A, Lubek JE, Papadimitriou JC, Basile JR, Younis RH (2017). Glycogen-rich clear cell squamous cell carcinoma originating in the oral cavity. Head Neck Pathol.

[12] Sheibani K, Battifora H (1988). Signet-ring cell melanoma. A rare morphologic variant of malignant melanoma. Am J Surg Pathol.

[13] Premalatha BR, Rao RS, Patil S, Neethi H (2012). Clear cell tumors of the head and neck: an overview. World J Dent.

[14] Yanofsky VR, Mercer SE, Phelps RG (2011). Histopathological variants of cutaneous squamous cell carcinoma: a review. J Skin Cancer.

[15] Kuo T (1980). Clear cell carcinoma of the skin. A variant of the squamous cell carcinoma that simulates sebaceous carcinoma. Am J Surg Pathol.

[16] Li TJ, Yu SF, Gao Y, Wang EB (2001). Clear cell odontogenic carcinoma: a clinicopathologic and immunocytochemical study of 5 cases. Arch Pathol Lab Med.

[17] Chu P, Wu E, Weiss LM (2000). Cytokeratin 7 and cytokeratin 20 expression in epithelial neoplasms: a survey of 435 cases. Mod Pathol.

[18] Khalid R, Ramanathan A, Tee Lun H, Lim D (2022). Aberrant expression of p63 in an adenocarcinoma of the prostate that has metastasized to the oral cavity. Cureus.

